# Draft Genome Sequence of CH_213, a Highly Cytotoxic Bacillus cytotoxicus Strain Isolated from Mashed Potatoes

**DOI:** 10.1128/MRA.00836-20

**Published:** 2020-08-27

**Authors:** Marc J. A. Stevens, Sophia Johler

**Affiliations:** a Institute for Food Safety and Hygiene, Vetsuisse Faculty, University of Zurich, Zurich, Switzerland; Queens College

## Abstract

Bacillus cytotoxicus CH_213 was isolated from a dehydrated mashed potato product purchased at a Swiss supermarket in 2017. The strain is closely related to strain NVH 391-98, which was linked to a foodborne outbreak of diarrheal syndrome in France in 1998.

## ANNOUNCEMENT

Bacillus cytotoxicus is a thermotolerant member of Bacillus cereus *sensu lato* and characteristically harbors *cytK1*, the gene encoding cytotoxin K (1). The species itself represents four phylogenetic clades, i.e., clades A to D; clades A and B contain strains that are more virulent than strains from clades C and D (2).

B. cytotoxicus CH_213 was isolated from mashed potato powder sample M16 in a study investigating the cytotoxicity of B. cereus *sensu lato* strains isolated from powdered food products (3). All other B. cytotoxicus strains characterized in that study exhibited either no or low levels of Vero cell cytotoxicity. In contrast, CH_213 exhibited very high cytotoxicity, 4.5-fold greater than the cytotoxicity of a highly toxic B. cereus outbreak strain (NVH 0075-95) used as a reference (3). Here, we present the draft genome sequence of this highly cytotoxic strain.

Chromosomal DNA was extracted from a single colony on brain heart infusion agar that had been cultivated overnight at 30°C using the DNA blood and tissue kit (Qiagen, Hombrechtikon, Switzerland). Paired-end sequencing libraries were prepared using the Nextera DNA Flex sample preparation kit (Illumina, San Diego, CA, USA). Sequencing was performed on an Illumina MiniSeq sequencer, which resulted in 870,759 paired-end reads of 150 bp, corresponding to an estimated coverage of 50-fold. The Generate FASTQ v2.0.2.17 module of the MiniSeq control software was used for read trimming. A quality check on the reads was performed using FastQC (Babraham Bioinformatics). The reads were assembled using Shovill v1.0.9, a software tool that uses SPAdes for genome assembly (4, 5). Standard settings were used, and the minimal contig size was 500 bp. The genome of CH_213 consists of 53 contigs, with a total length of 4,105,468 bp. The *L*_50_ value was 4, the *N*_50_ value was 234,866 bp, and the largest contig is 1,049,126 bp. The GC content is 35.6%. The genome of CH_213 was annotated using PGAP (6) and contains 4,234 genes, with 4,136 protein-coding sequences and 81 tRNAs.

Comparative genome analyses revealed that strain CH_213 is closely related to B. cytotoxicus strain NVH 391-98, which was isolated from vegetable puree and was the causative agent of a foodborne outbreak of diarrheal disease in France in 1998 that led to three fatalities (1). Single-nucleotide polymorphisms (SNPs) were determined using Parsnp v1.5.2 and revealed that strain CH_213 has 100 SNP differences compared to strain NVH 391-98. Comparison to strains in other B. cytotoxicus clades revealed at least 6,900 SNPs.

### Data availability.

Sequence and annotation data for B. cytotoxicus strain CH_213 were deposited in the GenBank database with the accession number JACCMP000000000. Reads were deposited in the Sequence Read Archive under the BioProject accession number PRJNA646186.
